# A dataset of annotated free comments on the sensory perception of madeleines for benchmarking text mining techniques

**DOI:** 10.1016/j.dib.2024.111250

**Published:** 2024-12-24

**Authors:** Michel Visalli, Ronan Symoneaux, Cécile Mursic, Margaux Touret, Flore Lourtioux, Kipédène Coulibaly, Benjamin Mahieu

**Affiliations:** a Centre des Sciences du Goût et de l'Alimentation, AgroSup Dijon, CNRS, INRAE, Université Bourgogne, F-21000 Dijon, France; b INRAE, PROBE research infrastructure, ChemoSens facility, F-21000 Dijon, France; c GRAPPE, ESA, USC 1422 INRAE, SensoVeg, SFR 4207 QUASAV, 55 rue Rabelais, F-49007 Angers, France; d Techni'Sens, 17000 La Rochelle, France; e Oniris, INRAE, StatSC, 44300 Nantes, France

**Keywords:** Open ended questions, Natural language processing, Sensory evaluation, Drivers of liking

## Abstract

This dataset was created to investigate the impact of data collection modes and pre-processing techniques on the quality of free comment data related to consumers' sensory perceptions. A total of 200 consumers were recruited and divided into two groups of 100. Each group evaluated six madeleine samples (five distinct samples and one replicate) in a sensory analysis laboratory, using different free comment data collection modes. Consumers in the first group provided only words or short expressions, while those in the second group used complete sentences. Additionally, participants reported their liking for each sample.

The collected data provided valuable insights into the effectiveness of the free comment method in sensory evaluation of food products. They emphasized the importance of data pre-processing and demonstrated how the chosen techniques can impact the quality of the results. The dataset is based on real-world consumer data, showcasing how individuals naturally express their subjective perceptions. It features descriptions that reflect authentic consumer language, including informal expressions, incorrect phrasing, spelling errors, and unstructured sentences. This raw textual data has been annotated and translated into English. The dataset can therefore be repurposed to assess and compare the performance of different text mining, natural language processing and sentiment analysis algorithms in both French and English, as well as to drive innovations in AI tools for sensory and consumer research.

Specifications Table

**Table:** 

Subject	Food science
Specific subject area	Sensory evaluation
Type of data	Table, Figure Raw, Processed.
Data collection	Two panels, each consisting of 100 French consumers, were selected and balanced as evenly as possible based on gender, age, household income, and frequency of madeleine consumption. These consumers were asked to evaluate six commercially available samples of madeleines (five distinct samples and one replicate). Using a computerized FIZZ questionnaire (version 2), they provided both liking scores (on a discrete scale ranging between 0 and 10) and sensory perceptions using the free comment method. Two modes of data collection were employed: consumers in the first panel responded using only words or short expressions (“FC words”), while those in the second panel were allowed to formulate complete sentences (“FC sentences”). The raw descriptions were manually encoded by two human operators using Excel. The first operator conducted the initial encoding, which was then reviewed and corrected by a second operator. The standardized descriptions adhere to a specific annotation format: “context word(s)/attribute/quantifier(s)”. Context words were encoded in nominal form, attributes in the masculine singular adjective form, and quantifiers in adverbial form. Sensory attributes having similar meanings were then aggregated into concepts based either on the interpretation of four human operators or the processing by two automated system (an expert system and ChatGPT). Both the raw descriptions, standardized descriptions and concepts were translated into English using Google Translate and subsequently reviewed and validated by the authors of this data paper.
Data source location	Consumers were recruited from the Techni'Sens database. The free comment and liking data were collected in the sensory booths at Techni'Sens in La Rochelle, which adheres to the NF-EN-ISO-8589 AFNOR standard.
Data accessibility	Repository name: Recherche Data Gouv Data identification number: 10.57745/6EAICO Direct URL to data: https://entrepot.recherche.data.gouv.fr/dataset.xhtml?persistentId=doi%3A10.57745%2F6EAICO
Related research article	

## 1 Value of the Data

• These data are valuable as they provide insights into the effectiveness of the free comment method for the sensory evaluation of food products.
• The dataset is grounded in real-world data, capturing how consumers naturally and subjectively express their perceptions. It includes descriptions that reflect the genuine expression of consumers, encompassing informal language, inappropriate phrasing, misspellings, and unstructured sentences.
• This unstructured textual data has been annotated, standardized, and accompanied by English translations. Researchers and developers can therefore reuse it to evaluate and compare the performance of various text mining and natural language processing algorithms across both French and English languages.
• The free comment data are associated with liking scores, making the dataset useful for training or refining sentiment analysis tools and models designed to detect affective content in consumer feedback. For example, transfer learning approaches can be used to enhance performances of sentiment analysis by leveraging pre-trained models for data augmentation [1].

## 2 Background

To date, few studies have examined the impact of data collection modes and pre-processing techniques on the quality of free comment data related to consumers' sensory perceptions. This dataset was collected to provide material for addressing this issue. However, the original research article limited its comparison to only three pre-processing techniques: manual processing by different human operators, automated processing using natural language processing, and automated processing using a large language model (ChatGPT). Nevertheless, this data article is valuable for advancing both a deeper understanding of methods of evaluation of sensory perceptions in consumer research and technical innovations in text mining.

## 3 Data Description

The dataset is provided in an XLSX file format, containing three datasheets.

Tab “Raw-Processed FC words”: Data collected and pre-processed from Group 1, where consumers responded using only words or short expressions.

Tab “Raw-Processed FC sentences”: Data collected and pre-processed from Group 2, where were allowed to respond using full sentences.

The two tabs are organized with the following columns:

- *product*: A 3-digit code representing the sample. Samples 416 and 971 are replicates of the same product.
- *consumer*: A code representing the consumer, prefixed with ‘W’ for consumers in Group 1 and ‘S’ in Group 2.
- *like_dislike*: ‘L’ if the free comment is associated with a positive perception, and ‘D’ if it is negative.
- *answer_number*: Ranges from 1 to 10 for FC words, and is set to 1 for FC sentences.
- *raw_description_fr*: The free comment reported by a consumer for a product (in French).
- *raw_description_en*: The English translation of “raw_description_fr”.
- *standardized_description_fr*: The result of the encoding of “raw_description_fr” by two human operators. The standardized descriptions adhere to a specific annotation format: “context word(s)/attribute/quantifier(s)”. Context words are nominal form words related to a perceptual dimension. Attributes are words in the masculine singular adjective form that describe a characteristic perceived in the product. Quantifiers are adverbs indicating the intensity of perception of an attribute or the level of appreciation for that intensity.
- *standardized_description_en*: The English translation of “standardized_description_fr”.
- *liking*: The score reported by a consumer for a product ranging from 0 to 10 that reflects the level of liking.

Tabs “Concepts”: Concepts extracted from Raw-Processed FC words/sentences by four human operators and two automated systems.

The two tabs are organized with the following columns:

- *operator*: “OP1-OP4” for human operators, “ES” for expert system, “LLM” for ChatGPT (see original research article for details)
- *product*: A 3-digit code representing the sample. Samples 416 and 971 are replicates of the same product.
- *like_dislike*: ‘L’ if the free comment is associated with a positive perception, and ‘D’ if it is negative.
- *consumer*: A code representing the consumer, prefixed with ‘W’ for consumers in Group 1 and ‘S’ in Group 2.
- *data_collection_mode*: ‘S’ for sentences, ‘W’ for words.
- *concept_fr*: Concept extracted by the human operators or the automated systems, in French.
- *concept_en*: The English translation of “concept_fr”.

**Fig. 1:**
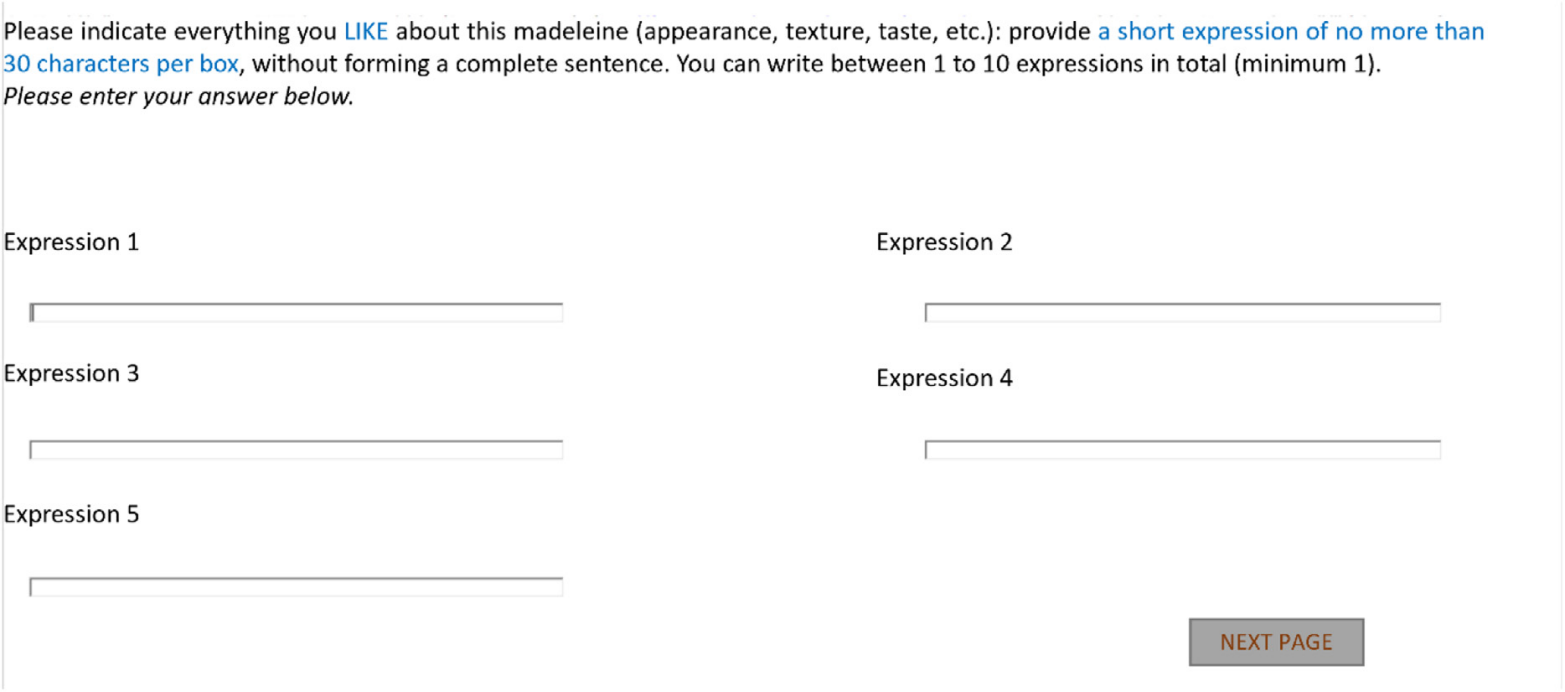
Data collection screen for Group 1 (translated from French).

Fig. 1 is a screenshot of the data collection screen for Group 1 (translated from French).

**Fig. 2:**
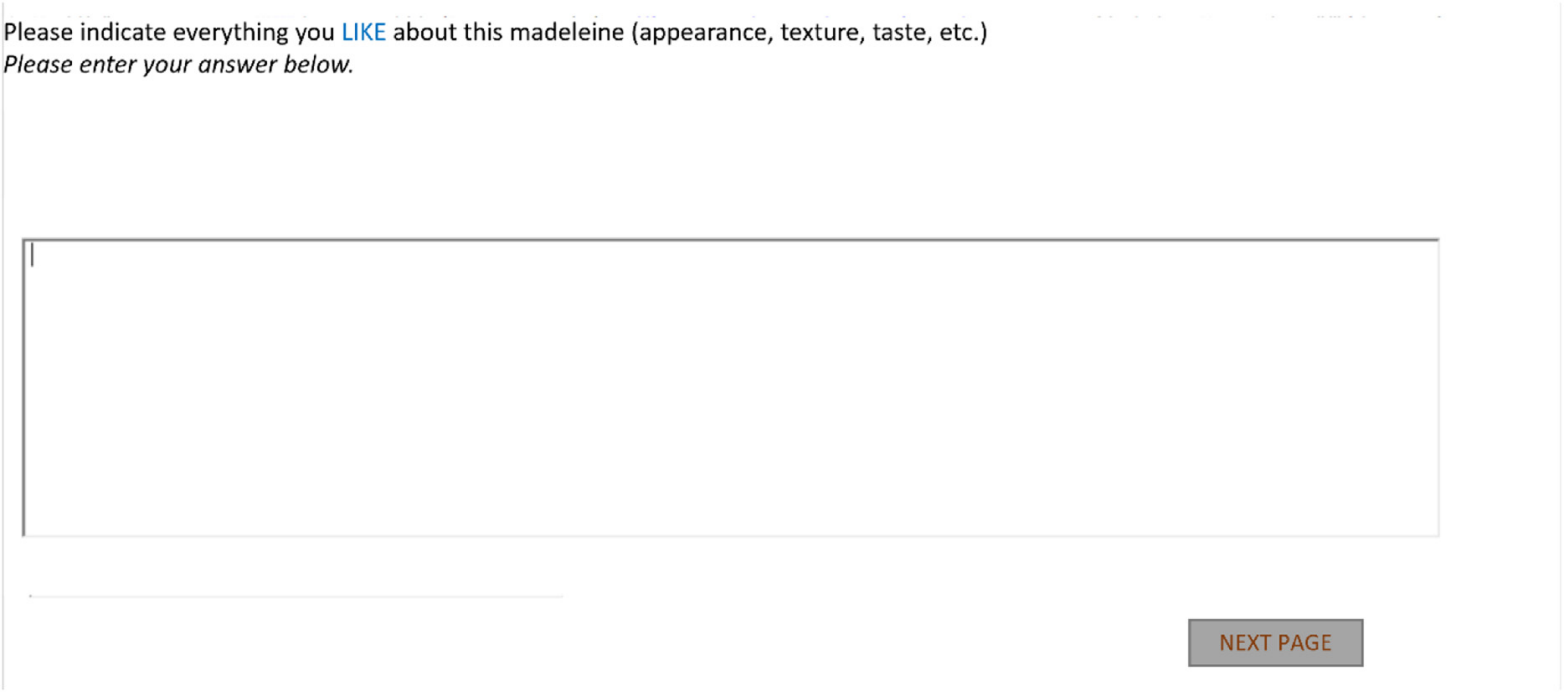
Data collection screen for Group 2 (translated from French).

Fig. 2 is a screenshot of the data collection screen for Group 2 (translated from French).

## 4 Experimental Design, Materials and Methods

A total of 200 consumers were recruited from the Techni'Sens database. Exclusion criteria included individuals who were pregnant, had food allergies, or did not consume madeleines. Eligible participants were contacted by phone to confirm their willingness to participate in the unpaid study and to validate their scheduled time. The consumer panel was split into two groups of 100, ensuring a balanced representation in terms of gender, age, household income, and frequency of madeleine consumption. The compositions of the groups are detailed in [2].

Participants were welcomed at the Techni'Sens sensory analysis laboratory.

The sessions were conducted during post-meal hours at 11 a.m., 12:30 p.m., 5 p.m., or 6:30 p.m. Participants evaluated six madeleines from five different commercial brands while seated in individual sensory evaluation booths equipped with computers running FIZZ software (version 2). Napkins were provided, and water was available throughout the duration of the session. The madeleines were presented to consumers in a sequential monadic order, following a William's Latin square design. They were served in their original individual plastic packaging, except for one madeleine, because of the visibility of the brand. Each product was assigned a random three-digit code, with one product presented under different codes (416 and 971). The compositions of the madeleines are detailed in [2]. Participants were instructed to rinse their mouths with water after tasting each sample. Each session lasted approximately 30 min.

The same evaluation procedure was applied to all six samples. Consumers were initially required to evaluate their liking for each madeleine using an 11-point discrete scale, with instructions stating: “On a scale of 0 to 10, please rate your overall liking of this madeleine.” Subsequently, on two consecutive screens, they were prompted to describe their likes and dislikes regarding the sensory attributes of the madeleines using the free comment method [3].

Two distinct data collection modes were used by consumers to report their free comments. Participants in Group 1 (“FC words”) were instructed to express their perceptions using separate answer boxes (min 1, max 10). The specific instructions were: “Please indicate everything you like (first and second screens, five boxes per screen)/don't like (third and fourth screens, five boxes per screen) about this madeleine (appearance, texture, taste, etc.): provide a short expression of no more than 30 characters per box without forming a complete sentence. You may submit between 1 and 10 expressions in total” (Fig. 1). In contrast, consumers in Group 2 (“FC sentences”) were free to use complete sentences, as no specific instructions were given regarding their responses (one answer box): “Please indicate everything you like (first screen) and don't like (second screen) about this madeleine (appearance, texture, taste, etc.)” (Fig. 2).

Two human operators encoded the raw free comment descriptions, with their characteristics outlined in [2]. They were directed to standardize the descriptions according to a specific annotation format: “context word(s)/attribute/quantifier(s)”. Context words were defined as nominal form words relating to a sensory modality (e.g., “texture”, “odour”, “smell”, “taste”, “appearance”, “visually”, etc.), indirectly relating to a sensory modality (e.g., “on the fingers”, “in-mouth”, “touch”, etc.), or relating to another perceptual dimension (e.g., “package”, “cooking”, “quantity”, etc.). Attributes were defined as words in the masculine singular adjective form (if possible; nominal form otherwise) that describe a characteristic perceived in the product (e.g., “sweet”, “fat”, “hard”, “soft”, “almond”, “shiny”, “yellow”, “artisanal”, etc.). Quantifiers were defined as adverbs relating to the intensity of perception of an attribute (e.g., “very”, “little”, “no”, “intensely”, “strong”, “slightly”, etc.) or to the level of appreciation of the intensity of an attribute (e.g., “too much”, “not enough”, “lack”, “excessive”, etc.). Examples of standardized descriptions: “taste/sweet/very”, “texture/soft/-“, “colour/beautiful/-”, “cooking/-/too”, “-/buttery/-“, “odour/-/”, “-/good/not”.

No subjective judgment was required from the operators when encoding the raw descriptions. The first operator performed the initial encoding, which was subsequently reviewed and adjusted by the second operator, both using Excel.

The standardized descriptions represent the verbatim information pertinent to describing the sensory perceptions of the madeleines. As such, they served as the “target” for evaluating the performances of pre-processing techniques presented in [2]. For reuse purposes, these descriptions can be utilized as annotations for training new models or for benchmarking various text mining techniques [4].

The concepts were determined either by a human operator or by an automated system. The level of detail of the concepts determines the grain at which FC data are subjected to statistical analysis. For human operators, the procedure was as follows. Each laboratory applied its own criteria and methods for concept extraction, with the two operators from each lab working independently. Concepts associated with “likes” were prefixed with “L_”, while those related to “dislikes” were prefixed with “D_.” All human operators used Excel and followed a four-step process: (i) dividing raw descriptions (if needed) to ensure each segment contained one attribute; (ii) categorizing these attributes within an explicit or implicit dimension when one or several contextual words were noted by the consumer; (iii) grouping synonymous attributes under a single concept after filtering based on the identified dimension; and (iv) associating quantifier groups with concepts when one or more quantifiers were mentioned by the consumer. Free comments about liking (e.g., “good,” “pleasant,” “bad,” etc.), irrelevant remarks (e.g., “none,” “nothing,” “all,” “nothing to report”), or unclear statements were excluded. More details about concept extraction can be found in [2].

The raw descriptions, standardized descriptions and concepts were translated into English using Google Translate. The authors of the article reviewed and corrected the translated results for accuracy.

## Limitations

The free comments were initially collected in French and subsequently translated into English. As a result, some original meanings may have been lost in translation. Additionally, any mistakes present in the French comments were mirrored in the English translations.

## Ethics Statement

As the objective of the study was to evaluate the sensory properties and preferences for commercially available food products, obtaining ethical approval from an institutional review board was optional in accordance with French law (n°2012–300 of March 5, 2012) concerning Research Involving Human Persons. The research was conducted in compliance with the Declaration of Helsinki. Participants were informed about the conditions of participation and signed a consent form

## CRediT authorship contribution statement

**Michel Visalli:** Data curation, Writing – original draft. **Ronan Symoneaux:** Writing – review & editing. **Cécile Mursic:** Data curation, Writing – review & editing. **Margaux Touret:** Data curation. **Flore Lourtioux:** Data curation. **Kipédène Coulibaly:** Writing – review & editing. **Benjamin Mahieu:** Writing – review & editing.

## Data Availability

Recherche.Data.GouvA dataset of annotated free comments on the sensory perception of madeleines for benchmarking text mining techniques (Original data).
